# PLLA Coating of Active Implants for Dual Drug Release

**DOI:** 10.3390/molecules27041417

**Published:** 2022-02-19

**Authors:** Katharina Wulf, Madeleine Goblet, Stefan Raggl, Michael Teske, Thomas Eickner, Thomas Lenarz, Niels Grabow, Gerrit Paasche

**Affiliations:** 1 Institute for Biomedical Engineering, University Medical Center Rostock, 18119 Rostock, Germany; michael.teske@uni-rostock.de (M.T.); thomas.eickner@uni-rostock.de (T.E.); niels.grabow@uni-rostock.de (N.G.); 2 Department of Otorhinolaryngology, Head and Neck Surgery and Hearing4all Cluster of Excellence, Hannover Medical School, 30625 Hannover, Germany; goblet.madeleine@mh-hannover.de (M.G.); lenarz.thomas@mh-hannover.de (T.L.); 3 MED-EL Medical Electronics, Fuerstenweg 77a, 6020 Innsbruck, Austria; stefan.raggl@medel.com

**Keywords:** PLLA coating, dual drug delivery, spiral ganglion neuron, impedance measurements, cochlear implant, diclofenac

## Abstract

Cochlear implants, like other active implants, rely on precise and effective electrical stimulation of the target tissue but become encapsulated by different amounts of fibrous tissue. The current study aimed at the development of a dual drug release from a PLLA coating and from the bulk material to address short-term and long-lasting release of anti-inflammatory drugs. Inner-ear cytocompatibility of drugs was studied in vitro. A PLLA coating (containing diclofenac) of medical-grade silicone (containing 5% dexamethasone) was developed and release profiles were determined. The influence of different coating thicknesses (2.5, 5 and 10 µm) and loadings (10% and 20% diclofenac) on impedances of electrical contacts were measured with and without pulsatile electrical stimulation. Diclofenac can be applied to the inner ear at concentrations of or below 4 × 10^−5^ mol/L. Release of dexamethasone from the silicone is diminished by surface coating but not blocked. Addition of 20% diclofenac enhances the dexamethasone release again. All PLLA coatings serve as insulator. This can be overcome by using removable masking on the contacts during the coating process. Dual drug release with different kinetics can be realized by adding drug-loaded coatings to drug-loaded silicone arrays without compromising electrical stimulation.

## 1. Introduction

Cochlear implants (CI) are currently the most effective treatment options for severe to profound hearing loss. During cochlear implantation, an electrode array consisting of different numbers of platinum contacts on a silicone carrier is inserted into the scala tympani of a cochlea. Cochlear nerve cells, the spiral ganglion neurons (SGN), can then be electrically stimulated by application of pulses of constant current. Clinical results with CI are typically good; for example, most patients can communicate via the telephone again [1]. Nevertheless, there are several known limitations. First, after hearing loss, SGN also start to degenerate [2]. Second, for insertion of a CI electrode, the cochlea has to be opened and the electrode array is positioned in the scala tympani. This causes some additional trauma which is considered to be a risk for surviving SGN [3]. As a reaction of the human body to this trauma, but also to the implanted foreign body, fibrous tissue is formed around the electrode array [4]. As shown in postmortem studies, the amount of tissue formation can be variable from a few cells to the formation of new bone [5]. The increase in electrical impedance at the stimulating contacts, as reported for the first two to three weeks after implantation [6], was shown to be correlated with the tissue response after implantation [7]. Furthermore, when the tissue formation is not uniform along the electrode array, it might also affect the specificity of the electrical stimulation.

Currently, there are several approaches under investigation to reduce trauma and the formation of fibrous tissue after cochlear implantation. Amongst them are surface patterning of the electrode array [8], application of drugs via pumps [9], cells [10], coatings [11], or from a reservoir, such as the silicone of the electrode array [12,13], as well as intraoperative deposition of steroids either directly [6] or by using a catheter [14]. To the best of our knowledge, besides one report on three patients receiving mononuclear cells obtained from bone marrow with the cochlear implant [10], only intraoperative deposition of steroids and steroid elution from the silicone of CI electrodes have been used clinically so far [12,14] and were shown to reduce or delay the impedance increase after implantation. As elution from the silicone results in a slow release [15], combination with a faster release from a surface coating might be a promising way to effectively address the tissue reaction right after implantation and in the long term.

The release of active substances can basically be divided into two types: diffusion-controlled drug release and chemically controlled drug release [16]. Diffusion-controlled release is further divided into membrane-associated and matrix-associated release. No matter which of these two is considered, both behave according to Fick’s first law of diffusion [16]. Characteristically, the release depends on the concentration gradient. At the beginning, the drug-release system is fully loaded whereas the tissue environment does not contain any drug. This results in a so-called initial burst release, a strong increase in concentration of the active ingredient in the tissue. In the further course of time, the release continues to level off.

In contrast, chemically controlled drug release requires steps that occur before the actual release. In so-called “swelling-controlled systems”, the active ingredient is distributed in a polymer matrix but cannot diffuse out of the material, e.g., due to a small pore size. After a solvent is added the polymer swells, causing the pores to enlarge in such a way that diffusion is no longer inhibited, and the active ingredient is released. In degradation-controlled systems, bonds have to be cleaved before the active ingredient is released. These bonds belong to the polymer in which the active ingredient is incorporated. Furthermore, there are systems in which the active ingredient is covalently bound to the polymer, e.g., as side chains. Hence, there is also a bond that has to be cleaved, before the active ingredient can diffuse out [17,18,19].

For dual drug release—which means the release of two active ingredients, each exhibiting a different release behavior—it is advantageous to use different mechanisms of release control. Therefore, drugs incorporated in polymer matrices can be used together with, e.g., drugs that are covalently immobilized at the surface of the dedicated polymer. Different polymers with different diffusion-controlled properties, e.g., different pore sizes, may also be used.

This principle was used to combine vascular endothelial growth factor (VEGF) and paclitaxel for application to the cardiovascular system [20,21]. There, paclitaxel was incorporated into a PDLLA polymer coating and VEGF was covalently attached to the surface of the polymer on either films or nanofiber non-woven. The resulting dual drug release improved endothelial cell viability in vitro, even in the presence of paclitaxel, which alone resulted in significantly decreased viability.

Furthermore, polymers with different diffusion properties can be used to obtain a dual drug release. For example, a silicone matrix can be loaded with an active ingredient that shows a relatively slow release. A faster-releasing coating on the silicone body then leads to either a faster release of the same or a different drug [15].

Dexamethasone (DMS) was already incorporated in coatings intended for CI [11,22]. Growth factors and other substances applied to the cochlea and being released from a coating were IGF1, HGF [23], BDNF [24], NT-3 [25] and Ara-C [22]. Most of these examples were intended to enhance the survival of SGN but not for reduction of fibrous tissue formation. A further search for additional substances that might reduce the inflammatory reaction and are already approved for other applications revealed diclofenac (DCF) [26,27] and enalapril [28,29] as possible candidates. DCF is a nonsteroidal anti-inflammatory drug. Its anti-inflammatory action can be explained by the inhibition of the cyclooxygenase in vitro and in vivo [30,31]. Enalapril is an angiotensin-converting-enzyme inhibitor and can reduce local inflammation after myocardial infarction [32].

Therefore, the aim of the current study was to investigate DCF and enalapril for their safety when applied to cells from the inner ear. In a second step, these substances should be included in a surface PLLA coating for a fast initial release and this PLLA coating shall be combined to DMS-loaded silicone of the electrode array for a slower long-term release of DMS. Release characteristics and the influence of the coatings on electrode contact impedances were investigated.

## 2. Results

### 2.1. Cell Culture

**Figure 1:**
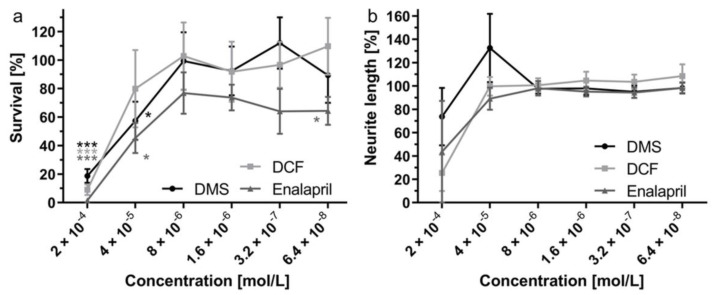
Survival (**a**) and neurite length (**b**) of freshly isolated SGN after addition of different concentrations of DCF, DMS or enalapril. * *p* < 0.05; *** *p* < 0.001 against controls. No differences between drugs were detected.

Diclofenac (DCF) and enalapril were tested regarding their effects on freshly isolated SGN in comparison to the known effects of dexamethasone (DMS). At concentrations of 2 × 10^−4^ mol/L, surviving SGN were barely found with all three substances (Figure 1a). Survival increased to about 100% at a concentration of 8 × 10^−6^ mol/L for DMS and DCF and remained stable for lower substance concentrations. After addition of enalapril, the highest survival of SGN with about 76.8% was achieved at a concentration of 8 × 10^−6^ mol/L. In contrast, neurite length was not affected for all three substances (Figure 1b). Here, only some reduction and fluctuations were observed at concentrations of 2 × 10^−4^ mol/L and 4 × 10^−5^ mol/L, where cell numbers were reduced. Based on the results it was decided to concentrate on DCF and DMS in further experiments.

### 2.2. PLLA Coating

**Figure 2:**
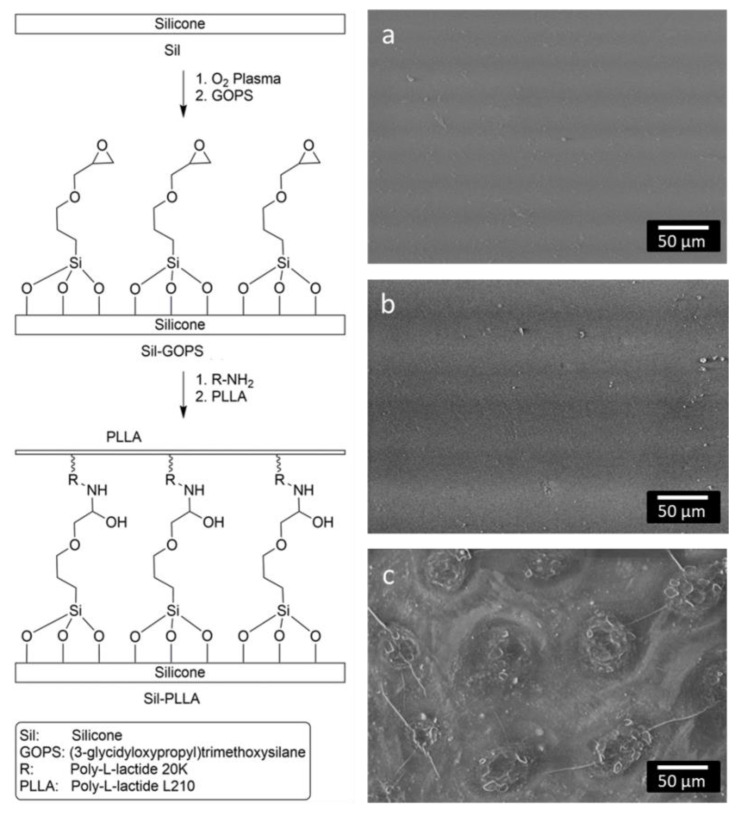
Left: General reaction scheme for the coating of silicone surfaces (Sil) with PLLA via the cross linker GOPS (Sil-GOPS) and the PLLA functionalized with amino groups (Sil-PLLA); Right: representative SEM micrographs of Sil (**a**); Sil-GOPS (**b**) and Sil-PLLA (**c**) surfaces.

In order to achieve a stable PLLA coating on silicone, the silicone surface (Sil) (Figure 2, left) was activated with O_2_ plasma and an intermediate layer of Silicone- (3-glycidyloxypropyl)trimethoxysilane (Sil-GOPS, Figure 2, left) was generated using the crosslinker GOPS and functionalized via PLLA-NH_2_. A stable PLLA coating resulting in Silicone- PLLA (Sil-PLLA, Figure 2, left) could be deposited on this intermediate layer.

In order to illustrate the surface morphology of the different layers, SEM images were taken. The Sil surface (Figure 2a) morphology only slightly changed with addition of GOPS (Figure 2b). However, with addition of the PLLA systems the modified Sil surface became more structured (Figure 2c).

### 2.3. Contact Angle Measurements

The hydrophilicity of the silicone surface changed with each reaction step as shown by the contact angles (Figure S2, Supplementary Materials). After addition of GOPS the contact angle decreased significantly, by about 50°. Due to the rather thin interlayer with PLLA-NH_2_, the measured contact angle changed only slightly. Afterwards, the surface was coated with high molecular weight PLLA and the contact angle again did not change significantly.

### 2.4. ATR-FTIR

**Figure 3:**
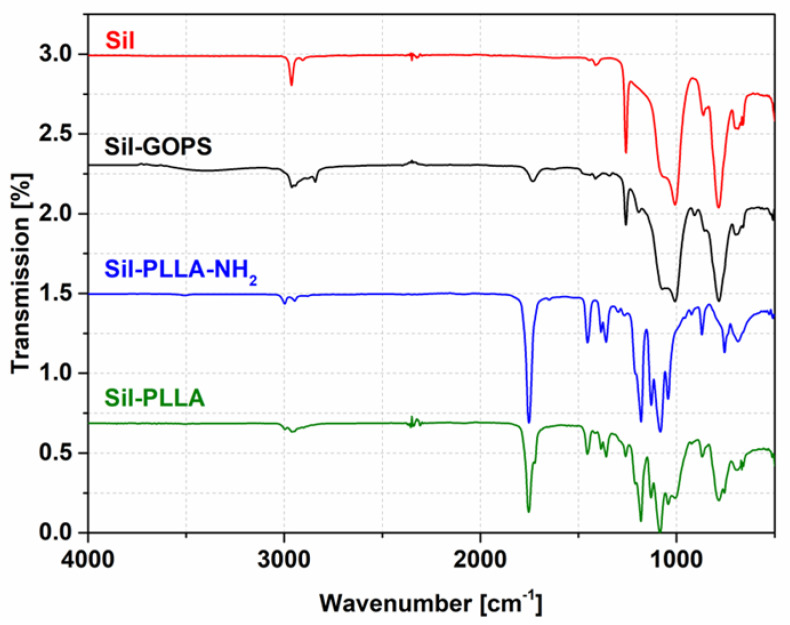
FTIR spectra of investigated Sil based samples Sil, Sil-GOPS, Sil-PLLA-NH_2_, Sil-PLLA in the range of 4000–500 cm^−1^. The prominent band between 1764–1712 cm^−1^ corresponds to PLLA signals.

In order to characterize the chemical changes on the Sil surface, the samples were analyzed by ATR-FTIR. The changes in characteristic IR bands indicate a chemical change in the surface composition. Significant differences in the range of 3500–3000 cm^−1^ and 1800–1700 cm^−1^ can be seen comparing the IR spectra Sil, Sil-GOPS, Sil-PLLA-NH_2_ and Sil-PLLA (Figure 3). Furthermore, the IR spectra for all PLLA coated silicone samples Sil-PLLA-NH_2_ and Sil-PLLA reveal a prominent band around 1751 cm^−1^ (Figure 3), which denotes carbonyl (C=O) stretching vibration, characteristic for ester bonds found in the used coating polymers. Furthermore, all FTIR spectra for the investigated samples were compared with the specific material used, shown in the supporting materials (Figures S3–S5, Supplementary Materials).

### 2.5. Drug Release

**Figure 4:**
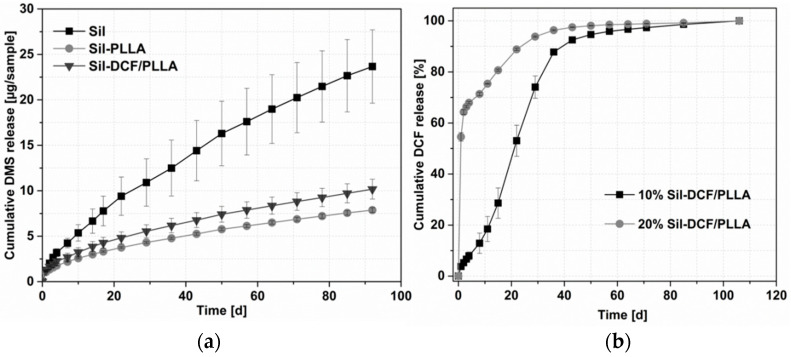
Cumulative in vitro release of incorporated substances from the samples with and without PLLA coating; (**a**): DMS release from uncoated and coated samples (Ø = 6 mm; DCF: PLLA 5: 95 wt%, *N* = 3) Release was with *p* < 0.05 significantly different between all investigated systems after 1 day. (**b**): DCF release from PLLA coated samples (DCF: PLLA 10: 90 wt% and 20: 80 wt%, *N* = 3). Release was with *p* < 0.05 significantly different between days 0 to 71.

As shown in Figure 4a, the DMS release is influenced by the addition of the PLLA coating. For the uncoated silicone samples, the released amounts of DMS are significantly higher compared to the coated samples. Around 24 µg DMS was released after 92 days, in contrast to the coated samples where 7 to 10 µg DMS was released in the same amount of time. Furthermore, the additionally incorporated DCF in the PLLA coating (Sil-DCF/PLLA) increases the DMS release significantly from day 1 compared to the other coated samples.

Besides the influence of the PLLA coating on the DMS release, also the release of DCF from the PLLA coating was characterized. In vitro release studies with 10 and 20% DCF in the coating were performed. As shown in Figure 4b, a significantly higher burst release was detected for the samples with 20% DCF content compared to the 10% samples. After only one day of release, more than 50% of DCF was already released from the 20% DCF-containing samples in contrast to the 10% DCF-containing samples with only 4% released DCF. With 10% DCF in the coating, it took about 20 days to release 50% of the DCF.

### 2.6. Impedance Results

**Figure 5:**
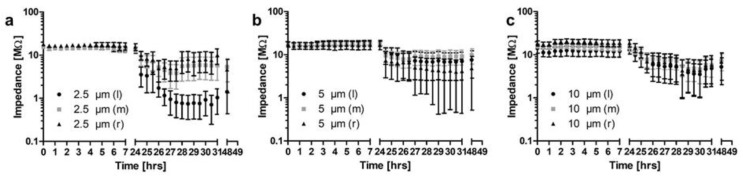
Mean (± SEM) impedance values as measured for contacts coated with PLLA at a thickness of 2.5 µm (**a**), 5 µm (**b**) or 10 µm (**c**). All measurements from *t* = 0 to 24 h were performed with the samples being immersed in 0.9% NaCl. Between all later measurements, left and right contacts were electrically stimulated. *N* = 5 each; l—left contact of the samples, m—middle, r—right contacts.

The influence of different PLLA-coatings with varying thicknesses (2.5; 5; 10 µm; *N* = 5 each) on electrical contacts was investigated using flat silicone samples, each having included three Pt-contacts comparable to cochlear implant electrode arrays (Figure 5a–c).

**Figure 6:**
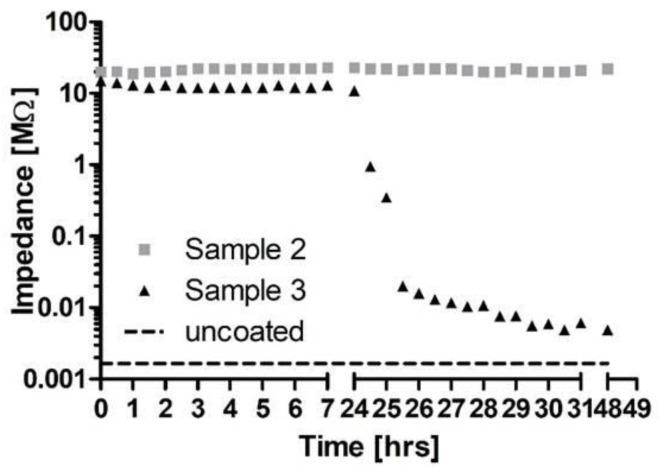
Impedance development over time on two contacts, both coated with 5 µm PLLA. Electrical stimulation started right after the 24 h measurement. The dashed horizontal line indicates mean impedance values of uncoated contacts (*N* = 6).

Initial impedances measured at 1 kHz were above 10 MΩ for 44 of the 45 contacts. The last contact showed impedances between 1 and 10 MΩ. Impedances for all contacts were stable during 24 h incubation in 0.9% NaCl. During the following 24 h of electrical stimulation of two of the three contacts (left and right contacts) on each sample, impedances of stimulated contacts were much more variable which is indicated by larger standard errors of the mean (SEM) in Figure 5a–c. Impedance could remain stable or drop to less than 10 kΩ (examples provided in Figure 6).

Average values for uncoated contacts (*N* = 6) were 1.65 kΩ and are indicated by a horizontal dashed line in Figure 6 for comparison. An overview on number of contacts and measured impedances after 24 h of electrical stimulation is provided in Table 1.

**Figure 7:**
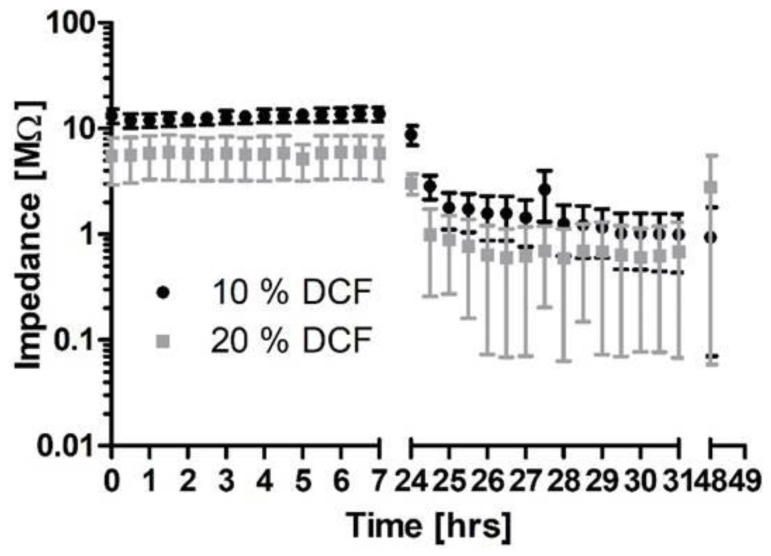
Mean (± SEM) impedance values as measured for contacts coated with PLLA and loaded with 10% or 20% DCF. All measurements from *t* = 0 to 24 h were performed with the samples being immersed in 0.9% NaCl. Between all later measurements, contacts were electrically stimulated (*N* = 8).

Similar results were found when 10% or 20% DCF were incorporated into a 10 µm PLLA coating (Figure 7). With 10% DCF, initial impedances were >10 MΩ for 11 out of 12 contacts whereas with 20% DCF, initial impedances were between 1 MΩ and 10 MΩ for 11 out of 12 contacts. For both concentrations, impedances could drop under electrical stimulation to below 10 kΩ (2/8 with 10% DCF and 4/8 with 20%) or remain at >1 MΩ (1/8 for both concentrations).

### 2.7. Effect of Electrical Stimulation on Coating

**Figure 8:**
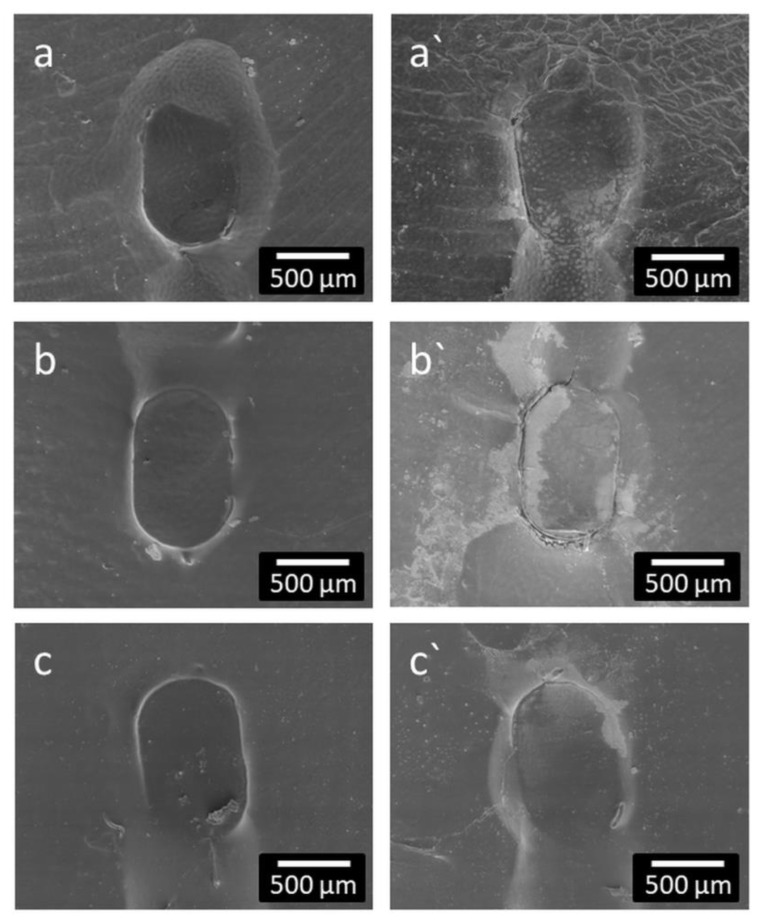
Representative SEM micrographs of platinum contacts after impedance measurements with intact (**a**–**c**) and damaged (**a`**–**c`**) PLLA coating of different thicknesses of 2.5 µm (**a**,**a`**); 5 µm (**b**,**b`**) and 10 µm (**c**,**c`**).

All samples were also examined for morphological changes after impedance measurements. As shown in Figure 8, for each coating thickness of 2.5, 5 and 10 µm, undamaged coating around the platinum contacts (Figure 8a–c) and platinum contacts with cracks and erosion of the coating (Figure 8a`–c`) were found.

### 2.8. Prevention of Coating of Contacts

In order to prevent the electrical contacts from being coated, masking was attached to the surface of the research electrode contacts prior to the PLLA coating process. After clearing the masked areas, impedances as measured at 1 kHz were between 5 and 24 kΩ (mean 14.0 ± 5.5 kΩ). When measuring the same contacts with the clinical system, impedances were between 2.71 and 4.13 kΩ (mean 3.31 ± 0.51 kΩ).

## 3. Discussion

Formation of fibrous tissue around the electrode carrier after implantation remains one of the challenges in cochlear implantation. This increases electrical impedances at the stimulating contacts and can reduce the specificity of the stimulation and therefore potentially compromise the hearing outcome with a CI. Potentially induced trauma to the cochlea during electrode insertion and also a foreign body reaction are considered possible reasons for the tissue reaction. To address these, the current project aimed at the combination of a long-term release of DMS from the silicone body of the implant and a short-term release of other suitable substances from a coating on the surface of the electrode array. DCF and enalapril were identified as suitable substances as both are considered to reduce the inflammatory reaction and are already approved for other applications [23,25].

### 3.1. Cell Culture

No substance applied to the inner ear should evoke toxic effects on spiral ganglion neurons. Therefore, both substances and DMS were first tested with freshly isolated SGN. Nearly no SGN survival at a concentration of 2 × 10^−4^ mol/L and a slightly reduced survival at 4 × 10^−5^ mol/L seem to indicate toxic effects at these concentrations. This can most likely be explained by the amount of solvent in the culture wells. DCF and DMS were dissolved in ethanol. Cell growth of HepG2 cells was strongly affected by an ethanol concentration of 2.5% [33], but the cytotoxic concentration differed depending on the cell type. As we had about 2% of ethanol in the samples with a substance concentration of 2 × 10^−4^ mol/L, we speculate that the reduced cell survival can be attributed to the amount of ethanol in the wells. At lower concentrations, no differences compared to controls were detected for addition of DCF. Therefore, application of DCF is considered safe at least at concentrations of 4 × 10^−5^ mol/L and below.

Enalapril was dissolved in DMSO. This led to a DMSO concentration of 0.28 mol/L in wells with an enalapril concentration of 2 × 10^−4^ mol/L. A slightly reduced cell survival can be expected in the range of 0.4 mol/L DMSO in the wells for fibroblasts and the solvent has to be considered toxic at a concentration of 0.7 mol/L [34,35]. Therefore, we expect that neuronal survival at 2 × 10^−4^ mol/L is most likely influenced by effects of the solvent. The DMSO concentration at 4 × 10^−5^ mol/L was 0.056 mol/L. Therefore, and according to the published results, cell survival should nearly be unaffected [34,35]. Addition of enalapril never resulted in SGN survival above 80%. As survival of SGN with addition of enalapril was also different to controls at more concentrations, its application to the inner ear was considered not safe. Therefore, in all further experiments the focus was put on DCF.

### 3.2. PLLA Coating

In order to reach a local long-term release of DMS and DCF, the DMS should be incorporated in the silicone carrier and the DCF should be embedded in a coating for the initial release. Coating silicone carriers is still a challenge due to their inert surface properties [36]. A desired PLLA coating of DMS-containing silicone without crosslinking resulted in a nonsufficient adhesion (data not shown). By first contact with water, the coating immediately peeled off. Therefore, a coating using the covalent binding of GOPS to silicone via the silane group and intermolecular forces between the PLLA-NH_2_-moiety and the PLLA-bulk was generated, with the PLLA-NH_2_ providing additional stability of the coating. The contact angle measurements revealed the alteration of the surface hydrophilic properties after each step of the coating. In contrast to the literature, contact angles after GOPS treatment remained quite high (80° vs. 57°) [37]. This goes back to the modification step. While the processes used in the literature are wet chemical in nature, our modification is a plasma-chemical process. The surface density of the groups generated by the O_2_ plasma for binding GOPS is thus lower than in wet-chemical processes. As a result, the silicone surface is not completely masked by the GOPS modification and still contributes to the contact angle. The slight increase in the contact angle after the binding of PLLA-NH_2_ was expected, because of the resulting thin PLLA-layer that presents a more hydrophobic surface. The contact angle after PLLA-coating remains the same as in the NH_2_-PLLA modification step as the material at the surface before and after the coating process remains PLLA. Chemical changes of the surface composition after each reaction step could also be confirmed by IR measurements. IR spectra of the polymer-coated samples exhibit several characteristic bands of the pure polymer as well as bands that correspond to the pure silicone.

### 3.3. Drug Release

Profiles of released DMS were detected for Sil, Sil-PLLA and Sil-DCF/PLLA. As DMS release decreases after PLLA-coating, the coating acts as diffusion barrier. Surprisingly, the incorporation of DCF in the PLLA coating led to a significant increase in the release of DMS from 7 to 10% after 13 weeks in vitro DMS release. This is likely due to the incorporation of DCF, which in turn leads to an altered spacing of the polymer chains of PLLA from each other, favoring the formation of enlarged pores compared to PLLA without DCF. To our knowledge, this is the first time that DCF sodium was incorporated in PLLA. Therefore, the behavior has also not been observed before, especially not in a dual drug-release system. The large increase in burst DCF release with 20% DCF in the layer compared to the 10% supports the hypothesis of enlarged pores by DCF in general and by the increased concentration in particular.

### 3.4. Impedance Measurements

As the function of cochlear or other active implants depends on precise electrical stimulation, and it is to be expected that any coatings of the electrode array will also be deposited on the stimulating contacts, the possible influence of the coating on electrical impedances was investigated. For this purpose, a setup was developed that allowed impedance measurements under reproducible and controlled conditions. The size of the stimulating contacts was chosen to be comparable to the clinically used Cl electrodes. As polymers, and especially the used PLLA, swell slightly by uptake of water after immersion in aqueous solutions [38], samples were just placed in physiological saline and impedances monitored for 24 h. Impedances were stable at very high values, indicating that the PLLA coating acts as insulator independently of the thickness of the coating. Parameters for electrical stimulation were adapted from Peter et al. [39]. The stimulating current was chosen to be safe for spiral ganglion neurons, at least in their in vitro setting. We can only speculate why impedances were reduced on some contacts with the electrical stimulation and on others, no change was detected. Furthermore, cracks in the coating after electrical stimulation were only found at contacts with reduced impedances and always at the transition from the contacts to the surrounding silicone. One possible reason could be the handling of the samples. Silicone is flexible whereas the Pt contacts are rigid. When samples were unintentionally bent during handling, tension within the polymer layer on the surface would be increased especially where the silicone meets the platinum. This could have introduced first cracks that facilitated water uptake and subsequently current flow. In addition, the influence of local voltage peaks at the edges of the Pt contacts cannot be excluded.

Interestingly, impedances of contacts with coatings containing 10% or 20% DCF were different by an order of magnitude right from the first measurement a few seconds after immersion in saline, but stable for at least 24 h. As shown, when there is 20% DCF in the polymer, more than 50% of it is released within 24 h. However, it is very unlikely that this amount of substance is released within a few seconds and the release remains halted for the rest of the first 24 h. Therefore, there must be different explanations for having lower impedances with 20% DCF. It might be that the high amount of the slightly hygroscopic DCF, which is incorporated in PLLA, leads to an increased and/or faster water uptake. This can result in altered spacing between the polymer chains of PLLA, favoring the formation of enlarged pores. Moreover, the increased concentration of the diclofenac sodium salt also increases the ion concentration within the coating, which may lead to increased conductivity and hence lower impedance.

As PLLA acts as insulator, strategies must be developed to avoid coating of contacts or to remove the coating from the stimulating contacts. For the current investigation, masking was additionally added on the contacts before coating. Removal of the masking after coating reliably reduced impedances at contacts as measured at 1 kHz. For a direct comparison with impedances of CI electrodes as measured with the clinical systems using rectangular pulses for the measurements, this approach was additionally taken for measurements of contacts where the coating was removed. Measured values were slightly increased compared to uncoated electrode contacts in the current setting and comparable to known impedance values of animal CI electrodes right before implantation [7] or commercial electrodes shortly after implantation [14].

## 4. Materials and Methods

### 4.1. Ethical Statement

The experiments with primary cells were conducted in accordance with the German “Law on Protecting Animals” (§4) and the European Directive 2010/63/EU for protection of animals used for experimental purpose, and registered (no. 2016/118) with the local authorities (Lower Saxony State Office for Consumer Protection and Food Safety (LAVES), Oldenburg, Germany).

### 4.2. Materials

The investigated silicone (Sil) and Pt-contact samples were provided by MED-EL (Innsbruck, Austria). All silicone samples used in this study contained 5 wt% DMS, which was added during the manufacturing process.

Poly-L-lactide (PLLA, L210) was purchased from Evonik (Schwerte, Germany). The crosslinker (3-glycidyloxypropyl)trimethoxysilane (GOPS) was purchased from Sigma-Aldrich (Taufkirchen, Germany) and PLLA-NH_2_ was provided by VWR (Dresden, Germany).

### 4.3. Preparation of Substances

Dexamethasone (Sanofi, Paris, France) and diclofenac (Sigma-Aldrich) were dissolved in ethanol (Carl Roth, Karlsruhe, Germany) whereas enalapril (Selleckchem, Munich, Germany) was dissolved in DMSO (#A3672 AppliChem GmbH, Darmstadt, Germany) at concentrations of 10 mmol/L. The stock solutions were further diluted with complemented cell culture medium to reach a concentration twice as high as the intended test concentrations.

### 4.4. Spiral Ganglion Cell Culture

Freshly isolated spiral ganglion cells were prepared from neonatal Sprague-Dawley rats (p3–5) following the protocol published by Schulze et al. [40]. In brief, after rapid decapitation, the cochleae were prepared from both halves of the skull and the bony shell of the cochleae was removed. After separating the spiral ganglia from the cochleae, these were enzymatically dissociated with HBSS containing 0.1% trypsin (Biochrom, Berlin, Germany) and 0.01% DNase I (Roche, Basel, Switzerland) for about 20 min followed by gentle trituration in 1 mL serum-free Panserin 401 (PAN Biotech, Aidenbach, Germany), supplemented with HEPES (23.4 µmol/mL, Invitrogen, Carlsbad, CA, USA), glucose (0.15%; Braun AG, Melsungen, Germany), penicillin (30 U/mL; Biochrom, Berlin, Germany), PBS (0.172 mg/mL; Gibco, Thermo Fisher Scientific, Waltham, MA, USA), N2-supplement (0.1%, Invitrogen, Carlsbad, CA, USA), and insulin (8.7 μg/mL; Biochrom, Berlin, Germany) until a homogeneous solution was achieved. Viable cells were counted and seeded at a concentration of 1 × 10^4^ cells per well in a 96-multiwell culture plate (TPP, Trasadingen, Switzerland), coated with 0.1 mg/mL poly-D/L-ornithine (Sigma-Aldrich) and 0.01 mg/mL laminin (natural from mouse, Life Technologies, Carlsbad, CA, USA). Cells were cultivated for 48 h in a mixture of complemented Panserin, supplemented with brain-derived neurotrophic factor (BDNF, Invitrogen, Carlsbad, CA, USA), and the different dilutions of the above-mentioned substances. The final BDNF concentration was 50 ng/mL and for the test substances 2 × 10^−4^ to 6.4 × 10^−8^ mol/L. Each concentration was tested 6 times (*N* = 6) with three repetitions (*n* = 3) on each plate. After 48 h, the cells were fixed by addition of a 1:1 mixture of acetone (J. T. Baker, Deventer, Netherlands) and methanol (Carl Roth) for 10 min and washed three times with PBS.

### 4.5. Immunhistochemistry

Following fixation, cells were incubated with the monoclonal mouse 200 kD neurofilament antibody (Novocastra, Leica, Wetzlar, Germany) for 1 h at 37 °C, 5% CO_2_. After rinsing with PBS, the secondary biotinylated anti-mouse antibody (Vector Laboratories Inc., Burlingame, CA, USA) was added for 30 min at room temperature before rinsing again with PBS. ABC complex solution (Vectastain^®^ Elite^®^ ABC-Kit, Vector Laboratories, Burlingame, CA, USA) was added to the cells using the protocol of the Vectastain^®^ Elite^®^ ABC Kit. Addition of diaminobenzidine (Peroxidase Substrate Kit DAB; Vector Laboratories, Burlingame, CA, USA) visualized the stained SGNs.

All surviving neurons of each well exhibiting a neurite length of at least three cell soma diameters were counted using a transmission light microscope (Olympus CKX41, Hamburg, Germany). For neurite length measurements, the five longest neurons in each field of view (one in the center and four around the perimeter of the well) were measured using the imaging software cellSens (Olympus, Hamburg, Germany) (compare Figure S6, Supplementary Materials). The survival rate was calculated by normalizing average cell numbers for each condition to average cell numbers in BDNF-treated control wells of the same 96-well plate before averaging across different plates. The same procedure was followed for evaluation of the neurite length. If not otherwise stated, values are presented as mean ± SD.

Statistical analysis of cell-culture results was performed by repeated measures ANOVA followed by Dunnett’s posttest using GraphPad Prism version 5.02 (GraphPad, La Jolla, CA, USA). *p* values of less than 0.05 were considered to be statistically significant.

### 4.6. Coating of the Silicone Surface

The cleaned silicone surfaces were activated via O_2_-plasma using 100 W power at 0.3 mbar for 1 min in a plasma chamber (Diener, Ebhausen, Germany). Then the samples were incubated in pure GOPS for 4 h at 90 °C. The activated samples were rinsed 3 times with ethanol and dried at 80 °C overnight under vacuum at 40 mbar.

The coating of the activated silicone samples was prepared via an established and characterized in-house manufactured spray-coating process. First, the activated silicone samples were spray coated with a thin polymer layer of PLLA-NH_2_ using a chloroform PLLA-NH_2_ (2 wt%) spray solution. Afterwards the samples were dried at 80 °C overnight and coated with pure polymer at thicknesses of 2.5, 5 or 10 µm (measured via microscopy (Olympus SZX16, Olympus, Hamburg, Germany)) or polymer/drug mixture, containing DCF to PLLA at ratios 10:90 wt% or 20:80 wt% in order to reach a layer thickness of about 10 µm. A chloroform PLLA (0.2 wt%) spray solution was used.

The surfaces were examined in a QUANTA FEG 250 (FEI Company, Germany) scanning electron microscope (SEM). A Contact Angle System (OCA 20, DataPhysics Instruments GmbH, Filderstadt, Germany) was used for analyzing surface modifications by contact angle measurements of ultra-pure water sessile drops. Presented mean values and standard deviations were calculated from *N* = 5 samples. Data were analyzed by Wilcoxon’s test using SPSS 27.0.

Attenuated total reflection—Fourier-transform infrared spectroscopy (ATR-FTIR)—measurements of the investigated silicone samples were performed using a Bruker Vertex 70 IR-spectrometer (Bruker, Ettlingen, Germany) equipped with a DLaTGS-detector. Each spectrum has been recorded in the range of 4000–500 cm^−1^ at a spectral resolution of 4 cm^−1^ and with 32 scans on the average using a Graseby Golden Gate Diamond ATR-unit (Bruker, Ettlingen, Germany). All spectra were analyzed using OPUS software (Bruker, Ettlingen, Germany) and were subsequently atmosphere and baseline corrected.

### 4.7. In Vitro Drug Release

The in vitro drug release studies were carried out under quasi-stationary and sink conditions. Between defined withdrawal time-periods each polymer sample (Ø = 6 mm) was left in the dark at 37 °C immersed in 1 mL artificial perilymph (145 mM NaCl; 2.7 mM KCl; 2 mM MgSO_4_; 1.2 mM CaCl_2_; 5 mM HEPES at a pH of 7.3). At the specific time, the elution medium was completely removed, replaced by 1 mL fresh artificial perilymph, and the drug concentration was quantified by HPLC. The chromatography was performed under isocratic conditions with a mobile phase consisting of acetonitrile/ultrapure water (50.5/49.5) (*v*/*v*), 0.15 M acetic acid and 4.7 mM trimethylamine at a pH 4.35. The flow rate was set to 1.0 mL/min. UV detection was conducted at a wavelength of 275 nm. The retention times for DMS and DCF are 3.8 and 8.3 min, respectively. MV and SD were calculated from *N* = 3 samples. In order to compare the release, the released amounts were normalized and referred to as the total amount of DMS (100%) and DCF (100%) in the samples. Data were analyzed by Kruskal-Wallis test using SPSS 27.0.

### 4.8. Impedance Measurements of Coated Samples

For impedance measurements of electrical contacts, flat rectangular silicone samples (1 cm by 1 cm) were generated (Figure S1, Supplementary Materials) with three Pt-contacts (approximate size: 480 × 800 µm) being fixed to one side of the sample. Two of these samples were placed in a beaker filled with 0.9% NaCl such that the samples were positioned approximately parallel at a distance of 2 cm with the contacts facing each other. Measurements were performed between one contact of sample 1 and one contact of sample 2 at 1 kHz by using a 3522-50 LCR-HiTESTER (Hioki, Ueda, Japan). One of the samples in the setup remained uncoated and served as reference electrode whereas the other was the test subject being either uncoated or having received one of the different coatings. Impedances were measured at room temperature without additional electrical stimulation after placing the samples in the beaker (*t* = 0), then every 30 min during the first 7 h and again 24 h after start. Pulsatile electrical stimulation (biphasic, pulse width: 400 µs, interphase gap: 120 µs, repetition rate: 1 kHz; current: 0.44 mA) was applied for the next 24 h to two of the three contacts (left and right contacts) by using a pulse generator (TGP 110, AIM-TTI, Huntingdon, UK). Impedance measurements were continued on all three contacts of the test sample according to the measurement regime of the first day. After the impedance measurements, the investigated surfaces were examined by scanning electron microscopy (SEM).

In additional measurements with coated research electrodes (MED-EL), the clinical MAESTRO software together with an attached MAX-box (MED-EL) was also used.

Data were analyzed using repeated measures ANOVA (two-way) followed by Bonferroni posttest.

## 5. Conclusions

After proving cytocompatibility of DCF for application in the inner ear and developing a coating strategy for drug-loaded medical-grade silicone, an approach to realize a dual drug release from cochlear implants was presented that combines a short-term release from a polymeric coating with a long-lasting release from the silicone body of the electrode array. The effect of the coating on electrode contact impedances was characterized and a strategy to overcome the insulation of the contacts was presented. The developed dual drug release for cochlear implant electrode arrays can now be investigated in vivo.

## Figures and Tables

**Table 1:** Overview on the number of contacts with very low or very high (unchanged) impedances after 24 h of electrical stimulation (*N* = 5 samples per condition with 2 stimulated contacts on each sample).

2.5 µm		5 µm		10 µm	
<10 kΩ	>1 MΩ	<10 kΩ	>1 MΩ	<10 kΩ	>1 MΩ
2/10	4/10	6/10	4/10	1/10	4/10

## Data Availability

All the data that support the findings of this study are available on request from the corresponding author.

## Supplementary Materials

The following supporting information can be downloaded online. Figure S1: Silicone sample as used for impedance measurements; (**a**): uncoated sample with three Pt-contacts and the connector; (**b**): Enlargement of one contact—coated version. Figure S2: Water contact angle ΘW ± standard deviation (SD) on silicone surfaces after each reaction step for sessile drop method (*N* = 5). *** *p* < 0.001. Figure S3: Fourier transform infrared spectra of investigated Sil-GOPS in comparison with pure GOPS in the range of 3500–500 cm^−1^; (**1**) prominent band at 1254 cm^−1^ depicts Si-CH_2_ bond from the GOPS structure (**2**) prominent band around 1254 cm^−1^ represents oxirane group from the GOPS structure. Figure S4: Fourier transform infrared spectra of investigated Sil-PLLA-NH_2_ in comparison with pure PLLA-NH_2_ in the range of 4000–500 cm^−1^; prominent band at 1751 cm^−1^ corresponds to the C=O stretching vibration from the PLLA structure. Figure S5: Fourier transform infrared spectra of investigated Sil-PLLA in comparison with pure PLLA in the range of 4000–500 cm^−1^; prominent band at 1751 cm^−1^ corresponds to the C=O stretching vibration from the PLLA structure. Figure S6: Microscopic image of stained spiral ganglion neurons (dark cell bodies) with 5 traced neurites (red). Treatment: 3.2 × 10^−7^ mol/L DCF. Scale bar: 50 µm.

## Sample Availability

Samples of the compounds used during the current study are available from the corresponding author on reasonable request.
