# Supplementary material for: PLLA Coating of Active Implants for Dual Drug Release
Source: Molecules. 2022 Feb 19;27(4):1417. doi: 10.3390/molecules27041417 (PMC8875406; doi:10.3390/molecules27041417)
Supplement: Supplementary file 1 [file molecules-27-01417-s001.zip › molecules-1604720-supplementary.pdf]

molecules

Article

PLLA Coating of Active Implants for Dual Drug Release

Katharina Wulf [1,*,†], Madeleine Goblet [2,†], Stefan Raggl [3], Michael Teske [1], Thomas Eickner [1], Thomas Lenarz [2], Niels Grabow [1] and Gerrit Paasche [2,*]

[1] Institute for Biomedical Engineering, Medical Center, Rostock University, 18119 Rostock, Germany; michael.teske@uni-rostock.de (M.T.); thomas.eickner@uni-rostock.de (T.E.); niels.grabow@uni-rostock.de (N.G.)
[2] Department of Otorhinolaryngology, Head and Neck Surgery and Hearing4all Cluster of Excellence, Hannover Medical School, 30625 Hannover, Germany; goblet.madeleine@mh-hannover.de (M.G.); lenarz.thomas@mh-hannover.de (T.L.)
[3] MED-EL Medical Electronics, Fuerstenweg 77a, 6020 Innsbruck, Austria; stefan.raggl@medel.com (S.R.)
* Correspondence: katharina.wulf@uni-rostock.de (K.W.); paasche.gerrit@mh-hannover.de (G.P.); Tel.:+49-381-54345520 (K.W.); Tel.:+49-511-5323808 (G.P.)
† These authors contributed equally to this work

Section: Materials and Methods Impedance measurements of coated samples

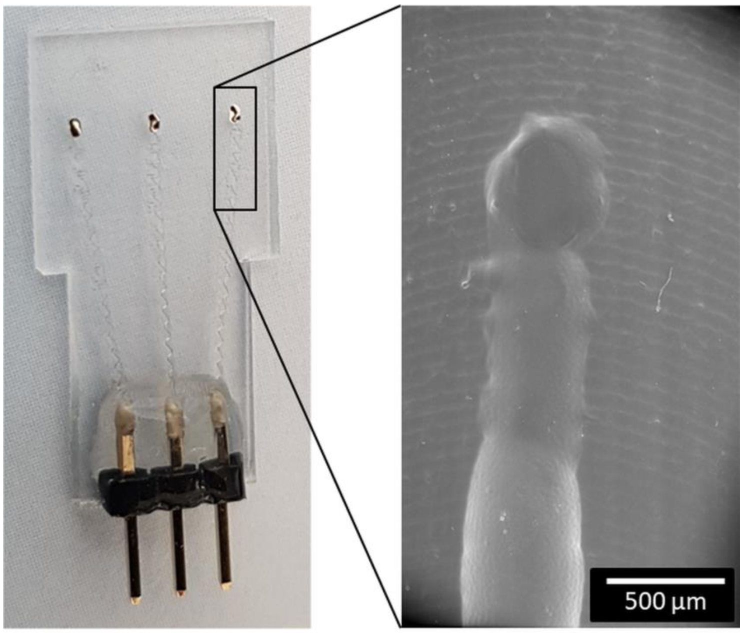

Figure S1. Silicone sample as used for impedance measurements; (**a**) uncoated sample with three Pt-contacts and the connector; (**b**) enlargement of one contact—coated version.

Section Results Contact Angle Measurements

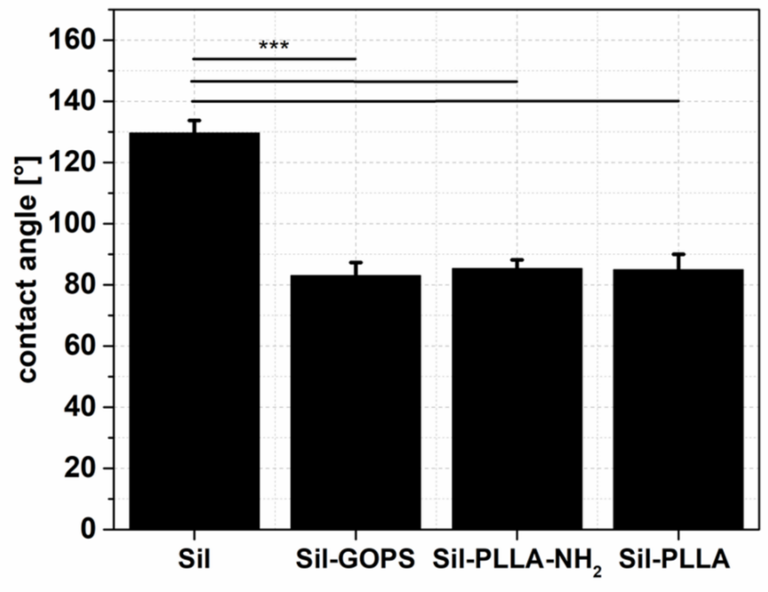

Figure S2. Water contact angle ΘW ± standard deviation (SD) on silicone surfaces after each reaction step for sessile drop method (N = 5). *** $p < 0.001$.

Section Results ATR-FTIR

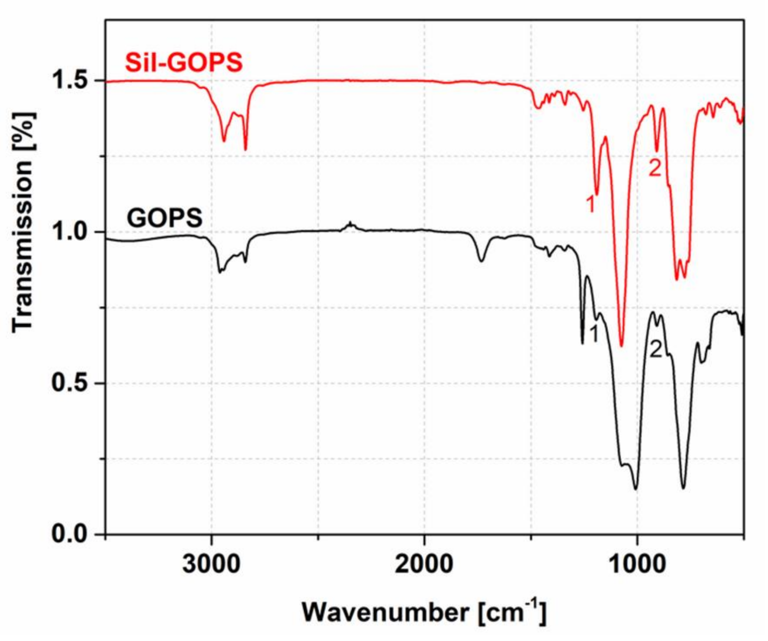

Figure S3. Fourier transform infrared spectra of investigated Sil-GOPS in comparison with pure GOPS in the range of 3500-500 cm^{-1}; (**1**) the prominent band at 1254 cm^{-1} depicts Si-CH_2 bond from the GOPS structure (**2**) the prominent band around 1254 cm^{-1} represents oxirane group from the GOPS structure.

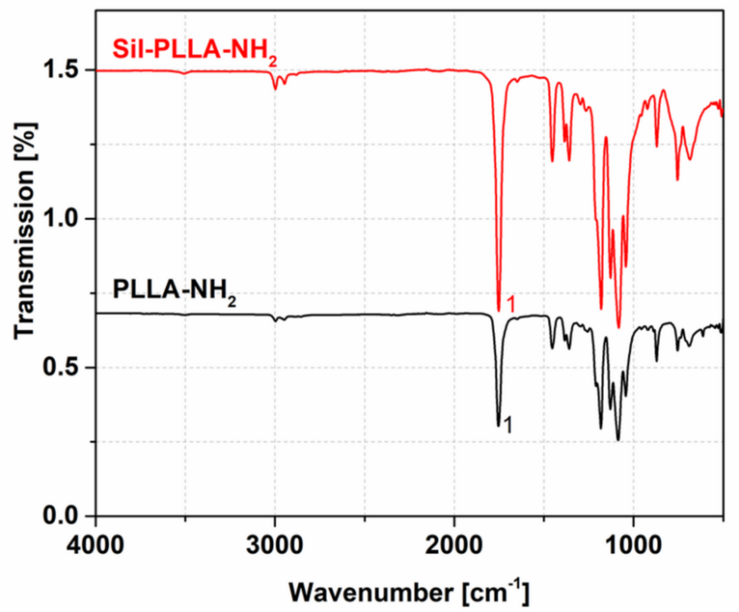

Figure S4. Fourier transform infrared spectra of investigated Sil-PLLA-NH_2 in comparison with pure PLLA-NH_2 in the range of 4000–500 cm^{-1}; prominent band at 1751 cm^{-1} corresponds to the C=O stretching vibration from the PLLA structure.

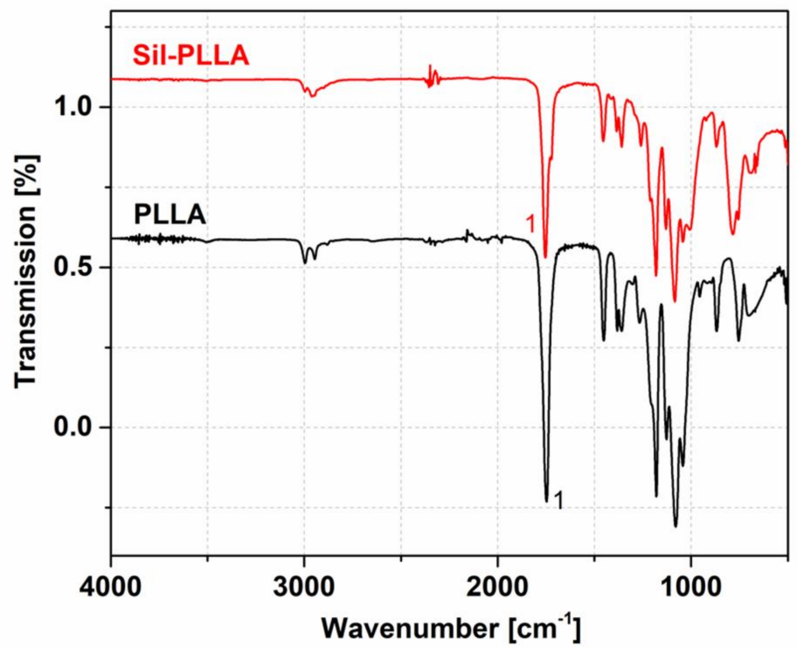

Figure S5. Fourier transform infrared spectra of investigated Sil-PLLA in comparison with pure PLLA in the range of 4000–500 cm^{-1}; prominent band at 1751 cm^{-1} corresponds to the C=O stretching vibration from the PLLA structure.

Section Materials and Methods Immunhistochemistry

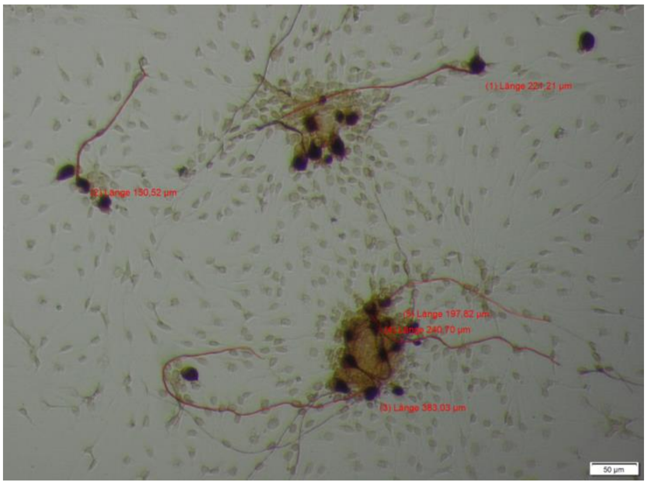

Figure S6. Microscopic image of stained spiral ganglion neurons (dark cell bodies) with five traced neurites (red). Treatment: 3.2x10^{-7} mol/L DCF. Scale bar: 50 µm.
